# Aerobic Exercise versus Electronic Cigarette in Vascular Aging Process: First Histological Insight

**DOI:** 10.1155/2023/8874599

**Published:** 2023-07-25

**Authors:** Vito A. Damay, Setiawan, Ronny Lesmana, Muhammad Rizki Akbar, Antonia Anna Lukito, Vita M. Tarawan, Januar W. Martha, J. Nugroho, Sony Sugiharto

**Affiliations:** ^1^Department of Cardiovascular Medicine, Universitas Pelita Harapan, Banten, Indonesia; ^2^Department of Biomedical Sciences, Universitas Padjadjaran, Bandung, Indonesia; ^3^Department of Cardiology and Vascular Medicine, Universitas Padjadjaran, Bandung, Indonesia; ^4^Department of Cardiology and Vascular Medicine, Universitas Airlangga, Surabaya, Indonesia; ^5^Department of Anatomical Pathology, Universitas Tarumanegara, Jakarta, Indonesia

## Abstract

Smoking is related to vascular aging. However, the hazardous effect of e-cigarette is often debatable, with limited studies available. In contrast, moderate-intensity aerobic exercise is well known to decrease aortic stiffness. We provide novel research to determine the effect of e-cigarette and aerobic moderate-intensity exercise on the aortic structure of Wistar rats. A total of 26 male Wistar rats (Rattus norvegicus) 8 weeks aged, 200-250 g b.w., were randomly divided into 4 groups, namely, K0 (normal rats), K1 (rats were given moderate-intensity aerobic exercise by animal treadmill 20 m/30 min), K2 (rats were given e-cigarette with 6 mg nicotine, 40% propylene glycol, and 60% vegetable glycerine 30 min for 5 days/week), and K3 (rats were given e-cigarette and moderate-intensity aerobic exercise). After exposure for 6 weeks, all animals were sacrificed to isolate the aorta for histopathological analysis with hematoxylin-eosin stain to evaluate the elastic fiber layer and intimal-medial thickness. The Verhoeff-Van Gieson staining was done for quantification elastic lamina fragmentation. Our study found that the e-cigarette group had the highest elastic lamina fragmentation among groups (8.14 ± 2.85). The exercise only group showed the lowest elastic lamina fragmentation (2.50 ± 1.87). Fragmentation in the e-cigarette and exercise group was higher than in the exercise only group (5.83 ± 0.753 vs. 2.50 ± 1.87, *p* = 0.002). There is a significant difference of NO serum between four groups. The result of post hoc analysis using LSD showed that there is a significant difference of NO serum between K0 and K2, K0 and K3, K1 and K2, and K1 and K3. Therefore, our research demonstrated that the most injury of aorta elastic lamina was in the group that was exposed to e-cigarette that leads to vascular aging while exercise is not yet proven to reverse this effect.

## 1. Background

Smoking is a significant risk factor for cardiovascular diseases (CVDs). CVDs are still the diseases with the highest mortality rate worldwide. Based on case reports from World Health Organization (WHO), at least 17.7 million global deaths annually are caused by these diseases [1]. Recently, many industries introduced an alternative to tobacco smoking called electronic cigarette (e-cigarette), considered safer than tobacco smoking.

People are changing from conventional tobacco smoking to e-cigarette, and even sport athletes nowadays are using e-cigarette [2]. In the past ten years, the use of e-cigarette by youth and adolescents has become more widespread, creating possible health risks. The incidence of past 30-day e-cigarette uses among high school students in the United States rose to 27.5% in 2019 before falling to 19.6% in early 2020 [3]. There is a strong promotional advertising that e-cigarette is safe for human health, which we know is still controversial. e-Cigarette has several chemical compounds such as nicotine, propylene glycol (PG), vegetable glycerine (VG), and several flavorings that cause endothelial damage elevated oxidative stress as part of vascular injury [4].

Despite the claim that e-cigarette is a safer alternative to tobacco cigarette, the toxic chemicals, similar to those found in tobacco cigarette, have been detected in e-cigarette aerosol [5]. Previous studies on animals and humans have found an increase oxidative stress as a result of e-cigarette usage [5]. The use of e-cigarettes results in the production of ROS, which in turn enhances the activation and expression of NADPH oxidase [5]. This process triggers the uncoupling of eNOS, leading to the formation of a harmful cycle of superoxide production and peroxynitrite formation [5]. This formation has been identified as a risk factor for atherosclerosis, myocardial ischemia, and hypertension [5]. The cycle results in protein modifications and depletion of BH4 [5]. The combined effect of these factors results in a reduction of NO, ultimately leading to impaired endothelial function [5]. A significant reduction of NO bioavailability was also exacerbated by longer exposure duration or higher aerosol nicotine levels [5]. Furthermore, e-cigarette exposure for 16 or 60 weeks has been found to contribute in superoxide generation with larger increases on a higher aerosol nicotine content [5]. The oxidative stress-induced eNOS uncoupling contributes to decreased endothelium-dependent vasodilator responses [5]. After 60-week exposure of e-cigarette on mice, over 18-fold increases of ACh dose are required for vascular relaxation [5]. It was concluded that the effects of e-cigarette exposure with onset of vascular endothelial dysfunction and eNOS impairment were much more severe than the effects produced by vascular aging [5].

Vascular aging remarkable mechanism is vascular stiffness. There have been multiple reports regarding the association between NO and vascular stiffness evaluated using pulse wave velocity (PWV) [6, 7]. The structural changes, however, remain poorly understood [7]. When interpreting raised PWV as a sole sign of increasing vascular stiffness, care must be used as blood pressure may confound the result [8]. Regarding the structural changes, internal elastic lamina fragmentation also contributes to wall fragility and decreases elastic strength which are needed to maintain the tension cause by flowing blood [9]. The maintenance of endothelial cell morphology has also been correlated with high eNOS expression induced by exercise based on previous study [9]. Reduced eNOS expression is indicative of deteriorated endothelial function [9]. Moderate-intensity aerobic exercise is widely known as a nonpharmacological treatment to improve vascular function and delay aging process.

Research about the exposure of tobacco cigarettes to vascular structure emergence is widely investigated, but the exposure of e-cigarette is still limited. Most of the research is through cell culture, or physiological biomarker and not on vascular structure histology. We conducted the novel research to explain the pathomechanism of vascular degenerative process directly on vascular structure histology that caused by electronic cigarette and how aerobic exercise delayed this aging process.

## 2. Methods

### 2.1. Study Design

This experimental study was conducted in the animal laboratory of the Faculty of Medicine Padjadjaran University, Bandung, Indonesia, from March 2022 to April 2022. The experimental protocol was approved by the Ethics Committee of Faculty of Medicine Padjadjaran University with an ethical serial number POB/14/KEP-UNPAD.

A total of 26 male Wistar rats (Rattus norvegicus) 8 weeks aged and 200-250 g b.w. were randomly divided into 4 groups, namely, K0 (normal rats), K1 (rats were given moderate-intensity aerobic exercise by animal treadmill 20 m/30 min), K2 (rats were given e-cigarette with 6 mg nicotine, 40% propylene glycol, and 60% vegetable glycerine 30 min for 5 days/week), and K3 (rats were given e-cigarette and moderate-intensity aerobic exercise). After exposure for 6 weeks, all animals were sacrificed to isolate the aorta for further histopathology analysis.

Wistar rats were housed in groups at room temperature of ±22-24°C, under a 12 : 12 h light-dark cycle, and allowed access to food and water ad libitum. All procedures were carried out according to established guidelines for the use and care of laboratory animals.

### 2.2. e-Cigarette Exposure Protocol

Exposure to e-cigarette used cigarette liquid or liquid Whale Dream produced by Pahlawan Lima Seven Inc., Jakarta, Indonesia, with a composition consisting of propylene glycol (40%), vegetable glycerin (60%), food flavoring, and 6 mg nicotine. The treatment group (K2 and K3) would be put into the smoking chamber. Cigarette liquid would be filled in the vape generator as much as 3 ml. The e-cigarette exposure process was carried out after preheating smoking chamber process for 15 minutes so that the exposure ran optimally. Treatment rats would be exposed to e-cigarette for 30 min/day, 5 days/week, for 6 weeks. Levels of carbon monoxide (CO) were maintained at levels of 250–300 ppm and CO_2_ at levels of 700–1200 ppm, using pollutant sensors. The tools were cleaned every 2 days so that the components contained in it were maintained. This protocol for e-cigarette exposure was modified from the study by El-Bestawy et al. [10] that had previously proven a shorter duration of 4 weeks; e-cigarette exposure had already influenced thoracic aorta.

The smoking room consisted of an aquarium glass with dimensions of 50 cm long, 20 cm high, and 30 cm high, e-cigarette generator room, generator room cover, fan, one-way valve, adapter, control box, aerator, ashtray, e-cigarette generator, and pollutant sensor. The size of the glass aquarium chamber was related to the number of rats that could be exposed to e-cigarette. The maximum number of rats that could enter the aquarium glass was 6-8 rats. The following formula is for determining the weight as soon as possible: Room volume = 50 cm × 25 cm × 30 cm = 37,500 cm^3^ = 0.038 m^3^ = 38 liters. The weight of the smoke with a density of air was 1.2 kg/m^3^, so the weight of the smoke in the box was 0.045 kg =45,000 mg.

(1) $$\begin{array}{c} \text{Part per million }\left(\text{ppm}\right)==\frac{45,000\text{ mg}}{38\text{ liter}}=\frac{1.184\text{ mg}}{\text{liter}}=1.184\text{ ppm}. \end{array}$$

The ventilation on the upper side of the 30 mm smoking chamber is aimed at maintaining the weight of e-cigarette smoke in the chamber so that it could be adjusted and maintained to maintain the desired level and prevent the rats from experiencing hypoxia. This smoking chamber device has obtained Intellectual Property Rights (IPR) from the Ministry of Law and Human Rights with a serial number EC00202222499 on April 5^th^, 2022.

### 2.3. Moderate-Intensity Aerobic Exercise Protocol

The physical exercise treatment group (K1 and K3) will be given adaptation training before the physical exercise intervention was carried out. Adaptation exercises were carried out for 2 weeks by running on a treadmill. During the first 1 week, it was used for adaptation in cage rooms to standardize their way of life and food before being subjected to physical activity treatment. The following week, the rats were introduced to the treadmill by training them to run on the treadmill every day at a gradually increased time, so that at the time the research was conducted, the rats had adapted to the conditions of the treadmill used. The introduction of this tool was carried out for 5 to 15 minutes and with a gradual speed until it reaches a speed of 20 m/s. In addition, during the adaptation period, rats would also be introduced to exposure to e-cigarette smoke first, which was for 10 minutes per day. At the same time as the fixed treatment group, the control group was kept in the smoking chamber but did not run on the treadmill and was not exposed to e-cigarette smoke.

At a speed of 20 meters/minute, blood lactate increased by 20 minutes after physical activity (sublactate threshold). At a speed of 25-30 meters/minute, blood lactate increased 2.5 times from baseline (supralactate or anaerobic threshold). After the adaptation exercise was completed, the rats were given moderate-intensity aerobic physical exercise at a treadmill speed of 20 meters/minute for 30 min/day, 5 days/week, for 6 weeks. This protocol of moderate-intensity aerobic exercise had been done previously by Sylviana et al. [11] and had been previously reviewed by Goenawan et al. [12].

### 2.4. Aorta Histopathological Assessment

Descendent thoracic aorta samples were fixed in neutral buffer formalin (NBF) 10%, processed to paraffin block, and then cut into 5 *μ*m thick sections. Slides were stained with hematoxylin-eosin to evaluate the vascular structure, number of elastic fibers, and intimal-medial thickness. The Verhoeff-Van Gieson staining was done to highlight the vascular elastin. Stain tissue section with working elastic stain solution for 15 minutes. Slides were rinsed in running tap water. The slide was then dipped in ferric chloride (2%) differentiating solution for 15 until 20 minutes and rinsed in tap water and sodium thiosulfate solution (5%) for 1 minute and in tap water for 2 minutes, followed by 2 changes in distilled water. Slides were stained using Van Gieson's solution for 2 minutes and rinsed twice with 95% alcohol. Dehydrate slides in absolute alcohol and mounted.

The histopathology of the aorta was observed using an Olympus CX31 microscope equipped with a 5-megapixel IndoMicro HDMI digital camera in which directly integrated with a computer device. Two anatomical pathologists (SS and PD) evaluated aorta histopathology preparations. Evaluation of aorta histopathology preparations was also carried out without knowing the treatment group. Each appraiser records the results obtained in the assessment result table form that had been provided. Some fiber elastic was manually counted, and intimal-medial thickness was using a microscope ruler. Elastic lamina fragmentation was manually counted based on breaks of elastic lamina in 4 random areas with 40x magnification.

### 2.5. Immunohistochemistry CD68 and NO Serum Evaluation

Immunohistochemistry of CD68 (Cell Marque, ready-to-use, USA) was done to evaluate the presence of macrophages or foam cells which is one of the main components in atherosclerosis. For NO serum evaluation, blood serum was obtained at the time of rat sacrifice. The unit measurement of NO serum is in micromolar (*μ*M). The NO concentration of the sample is calculated as follows:

(2) $$\begin{array}{c} \left[\text{Nitric oxide}\right]\text{ in }\mu \text{M}=\frac{{\text{OD}}_{\text{sample}}–{\text{OD}}_{\text{blank}}}{\text{slope}}, \end{array}$$

where OD_sample_ was the optical density value of the sample and OD_blank_ is the optical density value of water which was subtracted from the standard OD values and plotted against standard concentration. The slope was determined using linear regression fitting.

### 2.6. Statistical Analyses

All statistical analyses were performed using SPSS version 26 for Windows software. The Kolmogorov-Smirnov test was used to find the normality of the data. The alternative test was carried out using the Kruskal-Wallis method with a 95% confidence interval. The unpaired *T*-test and Mann–Whitney test were done to compare means between two treatment groups. The Pearson correlation statistic was done to get *r* value. Statistic means significant if *p* value < 0.05.

## 3. Results

### 3.1. Elastic Fiber Layers

**Figure 1:**
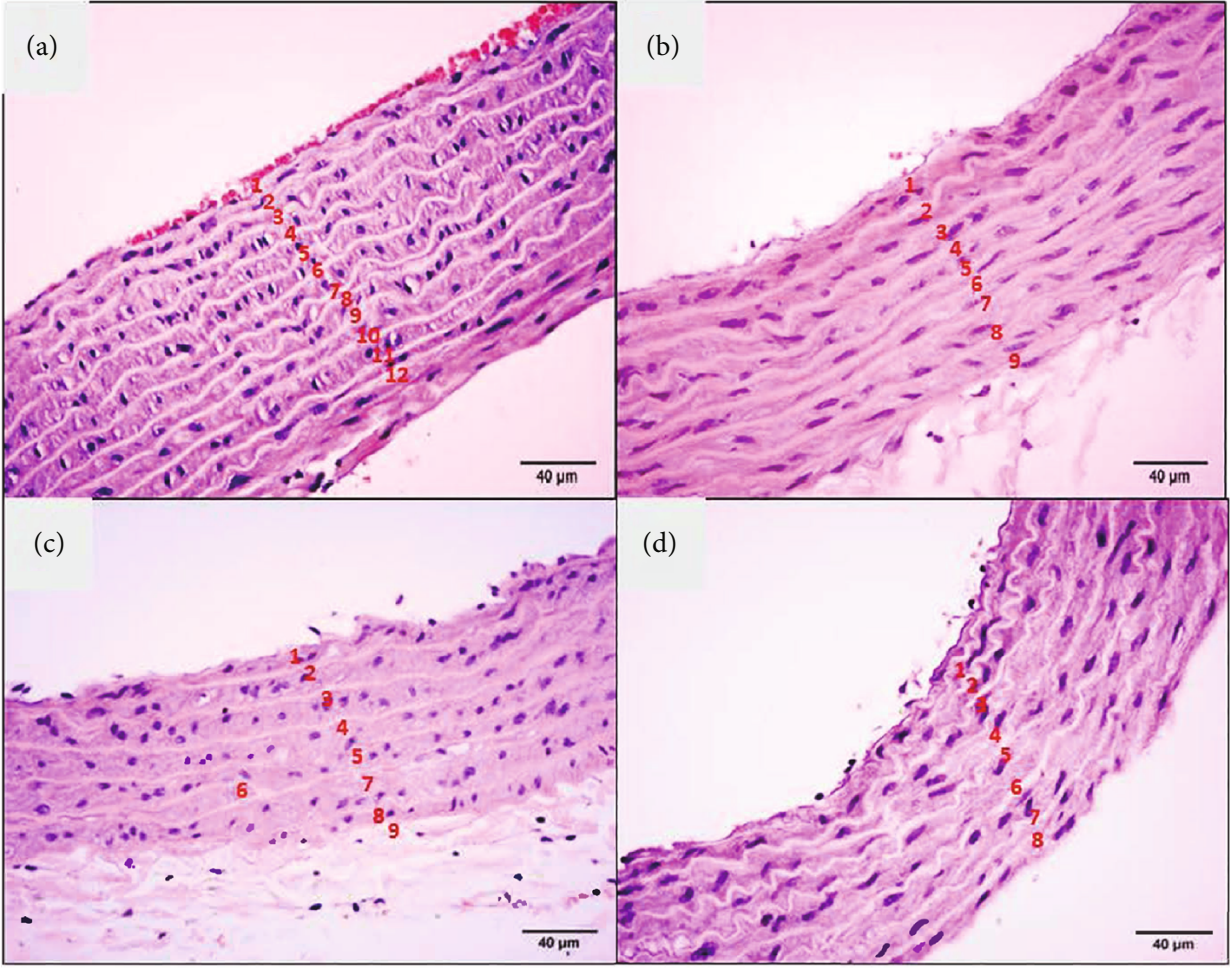
Elastic fiber layers. (a) Negative control (K0) with an average number of elastic fiber layers of 10.3. (b) Moderate-intensity aerobic physical exercise (K1) with an average number of elastic fiber layers of 9.5. (c) Exposure to electronic cigarettes (K2) with an average number of elastic fiber layers of 9.0. (d). The combination of exposure to electronic cigarettes and moderate-intensity aerobic physical exercise (K3) showed an average number of elastic fiber layers of 8.7 (H&E), 40x.

The negative control (K0) had an average number of elastic fiber layers of 10.3 ± 1.6, the moderate-intensity aerobic physical exercise group (K1) had an average of 9.5 ± 1.2, and the e-cigarette exposure group (K2) had an average of 9.0 ± 1.0. In contrast, the combination group of e-cigarette exposure and moderate-intensity aerobic physical exercise (K3) had an average of 8.7 ± 0.5 (Table 1 and Figure 1). The statistical analysis results related to the number of layers of elastic fibers showed *p* value > 0.05, meaning there was no statistically significant average difference in all treatment groups. The Mann–Whitney analysis between K2 and K3 also showed no significant difference (Table 2).

### 3.2. Tunica Intima-Media Thickness

**Figure 2:**
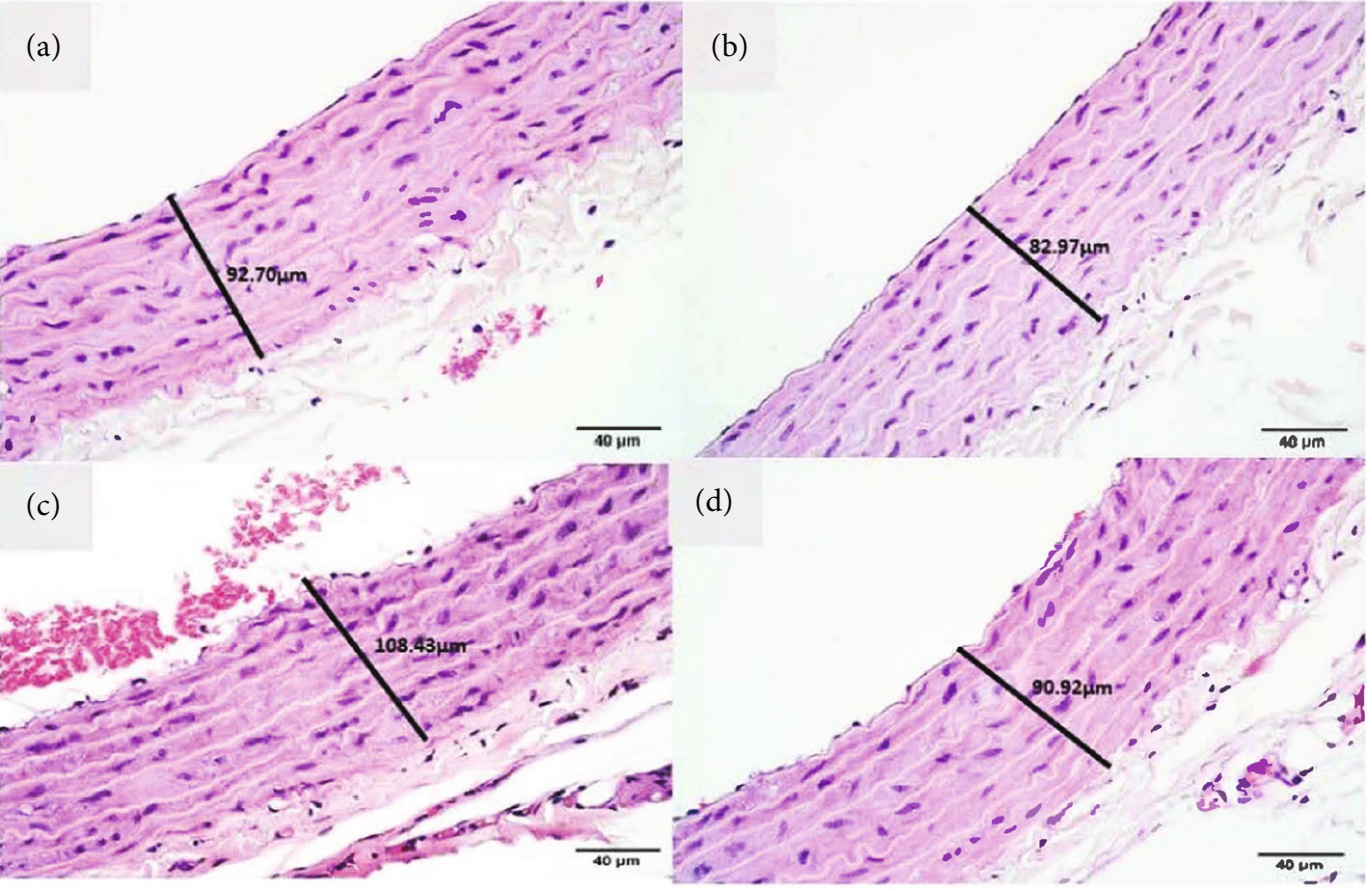
Tunica intima-media thickness. Average thickness of tunica intima-media (*μ*m). (a) The control group (K0) with an average of 104.8 *μ*m. (b) The moderate-intensity aerobic physical exercise group (K1) with an average of 95.6 *μ*m. (c) The e-cigarette exposure group (K2) with an average of 104.2 *μ*m. (d) The combination group of e-cigarette exposure and moderate-intensity aerobic physical exercise (K3) showed an average of 102.15 *μ*m (H&E), 40x.

The negative control (K0) had an average thickness of tunica intima-media of 104.8 ± 8.4, the moderate-intensity aerobic physical exercise group (K1) was 95.6 ± 5.1, and the e-cigarette exposure group (K2) was 104.2 ± 4.0. In contrast, the combination group of exposure to e-cigarette and moderate-intensity aerobic physical exercise (K3) had an average of 102.2 ± 3.4 (Figure 2). No significant differences were observed among groups (*p* value = 0.492) (Tables 3 and 4).

### 3.3. Elastic Lamina Fragmentation

**Figure 3:**
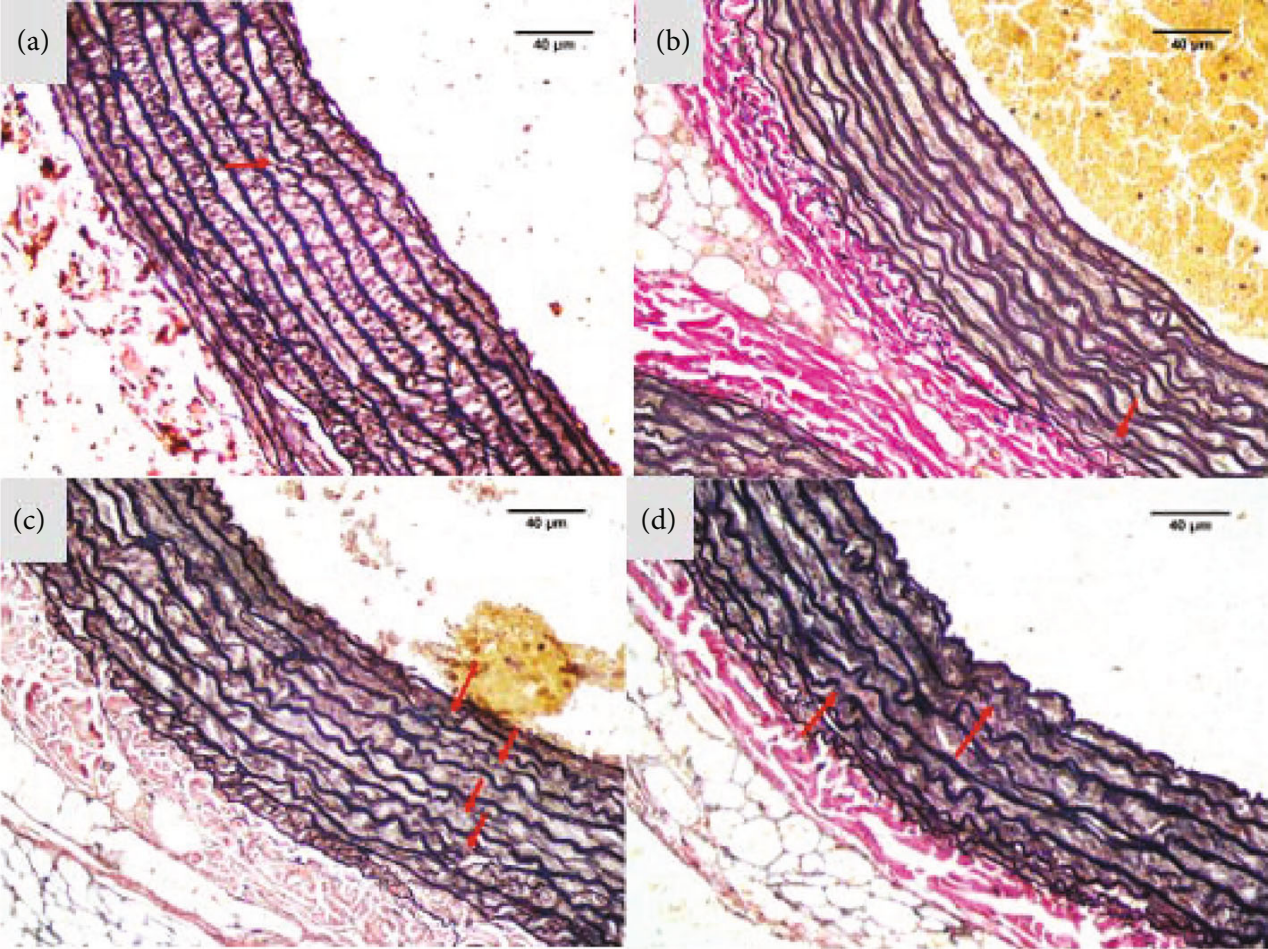
Elastic lamina fragmentation. (a) Negative control (K0) showed neatly arranged and dense elastic lamina with minimal fragmentation (red arrows). (b) Moderate-intensity aerobic physical exercise (K1) showed lower fragmentation than K0 (red arrows). (c) Exposure to e-cigarette (K2) showed increased elastic lamina fragmentation (red arrows). (d) Exposure to electronic cigarettes (K3) showed lower elastic lamina fragmentation (red arrows).

The elastic lamina fragmentation was analyzed based on the results of the Verhoeff-Van Gieson stain.  Table 5 shows the results of the Kruskal-Wallis test with a *p* value < 0.05, which means that moderate-intensity aerobic physical exercise has impact on changes in the vascular structure of the elastic lamina fragmentation variable with the Verhoeff-Van Gieson staining in all treatment groups. The control treatment group (K0) had an average elastic lamina fragmentation of 4.6 ± 2.4. The moderate-intensity aerobic physical exercise group (K1) had an average amount of elastic lamina fragmentation of 2.5 ± 1.9. The mean amount of elastic lamina fragmentation in the e-cigarette (K2) exposure group was 8.1 ± 2.9. The combination group of e-cigarette exposure and moderate-intensity aerobic physical exercise (K3) had an average amount of elastic lamina fragmentation of 5.8 ± 0.8 (Table 5 and Figure 3).

Statistical test results showed significant differences in the elastic lamina fragmentation variable between the K0 and K2 groups, K1 and K2 groups, and K1 and K3 groups, although there was no significant difference in the e-cigarette (K2) exposure group and the combination of e-cigarette exposure and moderate-intensity aerobic physical exercise (K3) (Table 6).

### 3.4. Immunohistochemistry CD68 and NO Serum Evaluation

**Figure 4:**
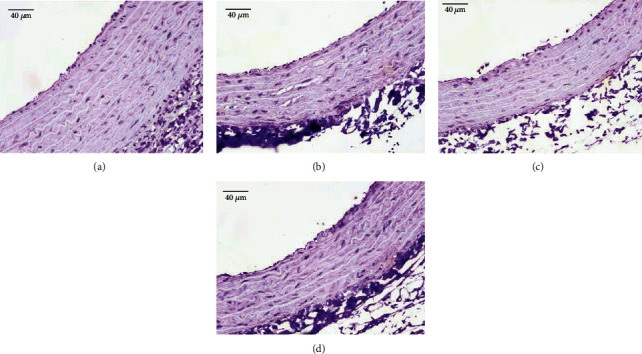
Immunohistochemistry CD68. The result of CD68 immunohistochemistry stains was found to be negative in all treatment groups (400x).

No CD68-positive macrophages were found in all groups, so it was assumed that no atherosclerotic lesions were formed (Figure 4).

NO serum for K0 is 44.8 ± 13.2, K1 is 55.4 ± 16.2, K2 is 31.1 ± 9.0, and K3 is 22.6 ± 9.1 (Table 7). Using one-way ANOVA, there is a significant difference of NO serum between four groups. Furthermore, a post hoc analysis with LSD was done (Table 8).

The result of post hoc analysis using LSD showed that there is a significant difference of NO serum between K0 and K2, K0 and K3, K1 and K2, and K1 and K3. There is no significant difference of NO serum between K0 and K1 and K2 and K3.

### 3.5. Correlation between NO Serum and Elastic Lamina Fragmentation

**Figure 5:**
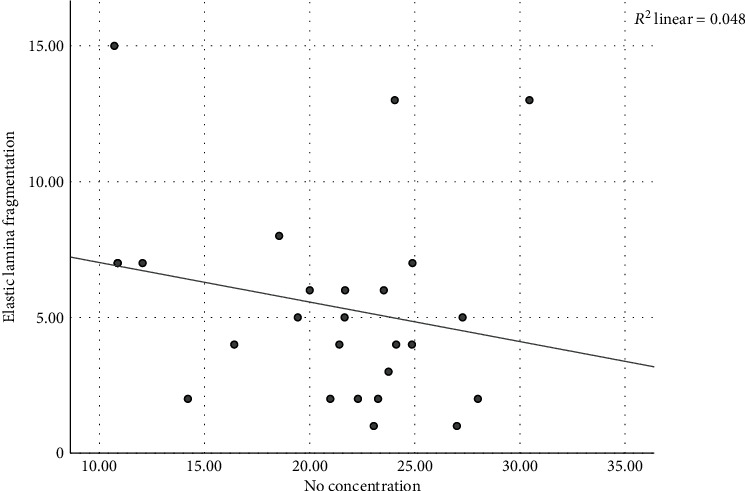
Correlation between NO serum and elastic lamina fragmentation. Scatter plot with fit line of elastic lamina fragmentation by NO serum showed negative correlation.

The Pearson correlation analysis showed that there is no statistical correlation between elastic lamina fragmentation and NO serum with *r* value of -0.218 and *p* value of 0.284. The negative trend showed that the higher the number of elastic lamina fragmentation, there will be a lower NO serum (Figure 5).

## 4. Discussion

### 4.1. e-Cigarette Affects the Vascular System

Research about the e-cigarette exposure is still limited. Currently, there has been no animal study evaluating the effect of e-cigarette exposure on vascular lamina although it is the hallmark of vascular stiffness. Previously published research only examined the foam cells or diameter of the vascular but did not observe the elastic lamina, which plays a main role in vascular stiffness. The study by El-Mahdy et al. [5, 13] had previously proved the effect of e-cigarette exposure on aortic stiffness from its histopathological preparation, but the assessment was done by looking the aortic thickness and not the elastic lamina fragmentation. There is no evidence yet in previous studies that study elastic lamina fragmentation may be the earliest vascular injury before atherosclerosis. Our study demonstrates the exposure effect of e-cigarette in the aorta of rats for 6 weeks. Chemical compounds inside e-cigarette, such as 6 mg of nicotine, 40% propylene glycol (PG), and 60% vegetable glycerine (VG), affect the histological structure of the aorta. Exposure to e-cigarette smoke for 4 weeks has shown no atherosclerotic lesions in tunica intima but alterations in smooth muscle cells (fragmentation and vacuolation) in tunica media but showed adverse effects on the intimal and medial layer and loss of endothelial cell integration and found many mast cells in the carotid arteries of rats. Previous study demonstrated that exposure to e-cigarette is composed of 18 mg/ml of nicotine, 70% of propylene glycol, and 30% of vegetable glycerine for 1 h/day, 6 days a week for 4 weeks, resulting in cardiac fibrosis. Exposure to cigarette smoke for 30 minutes a day, 5 days/week for 16 weeks, causes changes in the aortic wall, such as the elastic fiber count, elastic lamina fragmentation, deposition of pericellular matrix, and cell substitution and loss in the tunica media significantly [14–16].

We found no statistically difference in the number of layers of elastic fibers between groups. This result same with previous research about short-term exposure of electronic cigarette vapor does not cause significant alteration of tissue fibrosis [17]. The geometric and contractile properties of the heart are also not significantly affected by short-term e-cigarette vaping exposure [17]. In this study, the thickness of the tunica intima-media was not significantly different in all groups. According to the CD68 marker immunohistochemistry results, the protein expressed in monocytes showed no macrophages, which means there had not been an early stage of atherosclerotic lesions [18]. This study showed that one of the earliest defects in e-cigarette seen from histological observations is the elastic fragmentation of the lamina [18–20]. Therefore, the absence of CD68-positive staining is in fact supporting the evidence that electronic cigarette could have already caused elastic lamina fragmentation even before the early stage of atherosclerotic developed.

The group that was exposed to e-cigarette had higher fragmentation and significantly different from the group that only exercised; with the other groups, there was no difference in the meaning between the two groups [21, 22]. Due to lifelong mechanical loads and low-grade chronic inflammation, elastic fibers in the aorta wall gradually break as people age and lose their original elastic capabilities. The benefits of exercise are making vascular better and more elastic than those with e-cigarette alone. Exercise activity makes structure of the vascular significantly younger than those who exposed to e-cigarette [23, 24]. The role of the elastic lamina is to maintain the elasticity of blood vessels in the face of pressure and cardiac pumps, and its damage is related to the stiffness of blood vessels [16, 25]. Previous study by Park et al. [26] has found that regular participation in physical activity may mitigate the adverse effects of smoking on the vasculature. The study was done in young healthy men which conduct a treadmill run at least 2 hours per week for at least 6 months, and smoking status was defined as 8-10 cigarettes per day for at least 2 years. Our study was conducted in rat and did not find that exercise could be protective against aortic damage. The difference in our study despite the animal study design is that we have a shorter duration and we directly evaluate elastic lamina which might contribute to aortic stiffness in the future. Despite a shorter duration in our study, previous study has found that an even shorter period of treadmill exercise of 4-week duration with 30-minute run and 3 days per week could already reduce aortic stiffness according to PWV measurement [27]. Thus, our study might indicate that even after conducting a moderate-intensity aerobic exercise for 6 weeks, this could not mitigate the harmful effect of smoking e-cigarette especially for its influence on elastic lamina fragmentation.

Vascular stiffness also occurs in the aging process and hypertension. The process of vascular aging showed an increasing of elastic fragmentation that is the decrease in elastin content and the increase in collagen content [28]. Age-related changes in vascular diameter size were identified. Different changes occurred in the composition of each tunica layer within the artery walls, such as the irregular shape of endothelial cell, increasing of collagen fibers, and fewer smooth muscle cells [29]. Additionally, it was found that the elastic fibers diminished and began to fragment, particularly in the aorta. Numerous literature studies have discussed about mechanism of elastic fiber alteration with age as the aorta alters in different places and the elastic fibers in the tunica media of the aortic wall age. Elastic fibers in the tunica media degenerate as people age, and elastin fragmentation is common. The aorta is less elastic in the elderly than in the young [30].

There was an association about chronic smoking in sedentary men, especially increase of aortic stiffening. Moreover, significant smoking-induced increase in aortic stiffness was not found in physically active adults. Smokers appear to have vascular stiffness based on pulse wave velocity (diagonal line) examination. People who is physically active can minimize vascular dysfunction. The difference is that the effect on blood vessels is observed by examining the pulse wave velocity. This examination can assess the vascular stiffness. The study assessed that someone who smokes and is physically inactive has stiffer blood vessels than those who do not smoke and are physically inactive [26]. Smoking harms the vascular system, but there was no difference in the vascular stiffness in the active sports group, both smokers and nonsmokers [19, 20, 26]. The difference in our study with the Wistar rat experiment was directly observing the structure that contributes to the stiffness of blood vessels, namely, the elastic lamina. Other factors cause elastic lamina damage, including obesity, aging, and genetics. The previous study demonstrated that obese mice have a reduced fenestra number in the elastic lamina in mesenteric arteries, which leads to aorta stiffer [31]. On the other hand, aging has been known as an affecting factor in the vascular elasticity, loss of interlaminar crosslinks. This case indicated that aging is accompanied by destroying the elastic vascular structure [32, 33]. The previous studies also showed that rats with aging (aged 1.5–2 years) have thin, fragmented, and destructed elastic lamina compared with adult rats (aged 3–4 months) [34, 35]. The degradation of elastic fibers caused by aging or loss by damage would not regenerate spontaneously and affects the biomechanical properties of the aorta wall. Aging process in vascular indicates the alteration of ECM and vascular stiffness. Previous study demonstrated that carotid stiffness was elevated in aging mice and associated with reduction of elastin as an essential protein in media [28].

Nicotine inside e-cigarette can enter our body and stimulate the immune system, which leads to an inflammation process mediated by the transcription factor Nf-k*β* [30]. This toxin will induce elastin fragmentation leading to aortic damage and irreversibly increasing aortic stiffness [36, 37]. Furthermore, propylene glycol and vegetable glycerine also impact blood vessels because heating methods from the device result in carbon monoxide (CO), polycyclic aromatic hydrocarbons, and heavy metals which induced endothelial inflammation and increase in adhesion molecules such as intercellular adhesion molecule-1 (ICAM-1) and tumor necrosis factor-*α* (TNF-*α*) [38]. All of the molecular alterations in cellular would affect structural changes, especially aorta histology [39]. Nicotine raises blood pressure and associated with vascular alteration in both humans and animals. Nicotine alone can cause vascular dysfunction, as shown by the fact that nicotine-treated animals displayed impaired endothelium-dependent relaxation to acetylcholine compared with control rats and that the arterial response to phenylephrine-induced contraction is greater in nicotine-treated rats than in control rats [39, 40].

We also proved that exposure of e-cigarette caused elastin damage, which plays an essential role in vascular stiffness as in previous studies, which was proven by pulse wave examination in the previous study but has never been proven histopathologically like in our study. Our research demonstrated that e-cigarette caused a significantly higher elastic lamina fragmentation suggesting a faster degenerative process, and thus, this might contribute to vascular stiffness in the future.

We found that e-cigarette significantly reduces NO serum. This result is consistent with previous findings [5]. Moreover, the effect of e-cigarette on NO is much faster compared with the previous study by El-Mahdy et al. [5] with only 6 weeks based on our experiment. Our result supports the fact that e-cigarette alters NO bioavailability due to activation of NADPH oxidase by ROS leading to uncoupling of eNOS [5]. The reduction of NO serum was also consistent with the result regarding the elastic lamina fragmentation [5]. As previously suggested, there was a correlation between the expression of eNOS and the preservation of the morphology of endothelial cells [5]. Therefore, our study is also consistent supporting that the manifestation of impaired aortic structure, in this case, elastic lamina fragmentation, could be inferred from the diminished expression of NO. Additionally, our result also showed that the protective role of exercise that supposed to increase NO has been failed due to the exposure of e-cigarette. Instead of at least preserving the NO serum, it is found that the group with e-cigarette still significantly has a reduced NO serum even after exercising. Our result may suggest the deleterious effect of e-cigarette which could potentially abolish the protective effect of exercise that had been done.

### 4.2. Effects of Exercise on Vascular System

In our experiment, the exercise only group has less fragmentation on elastic lamina than the group with exercise and e-cigarette. This means that the benefits of exercise could be reduced by vascular injury caused by e-cigarette exposure. Previous study demonstrated that aerobic exercise could decrease aorta stiffness in obese mice [41]. The activity of exercising alone would be better if not exposed to e-cigarette. Moderate-intensity aerobic exercise 5 times/week for 20 minutes of running might elevate oxidative stress levels that measured by malondialdehyde (MDA) levels. Moderate-intensity aerobic physical exercise increases reactive oxygen species in the abdominal aorta tissue. Exercise is a physical stressor that can disturb homeostatic balance of the body. Thus, it will increase reactive oxygen species (ROS) [24]. The aerobic exercise group only has less elastic lamina fragmentation compared with the e-cigarette group only and the group with exercise and e-cigarette. This also means that the structure of the exercise group only was better than the group exposed to e-cigarette [35]. Previous study has showed that exercise reduced MMP-2 and MMP-9 expressions in aortic tissue, decreased elastic fiber fragmentation in aortic wall, and also improved aortic wall elasticity [28].

Exercise activity, especially endurance training, causes many adaptive changes which mostly affect skeletal muscles. These adaptations involve modifications to tissue composition and metabolism [42]. Extracellular matrix (ECM) adaptations take place concurrently. ECM interacts with cells to communicate information, which influences cellular development, adhesion, and differentiation as well as tissue reproduction. It also serves as a framework and structural support for cells and organs. Skeletal muscle ECM remodeling affects cellular functions, such as DNA synthesis, fragmentation of microtubules, and fusion of myoblast. This remodelling resulted in increase of muscle strength, elasticity of tissue, and damage resistant [15, 42]. The regeneration of muscle fibers also involves the ECM. The ECM-modifying enzymes can induce abnormality in muscle regeneration process. There are several contributors to the remodelling of elastin as an essential of ECM components that play important role for tissue elasticity, including elastase, cathepsin K, and plasmin. Additionally, proteolytic enzymes can have a direct impact on muscle fibers by, for example, causing apoptosis [25]. Impaired of assembly or other disruption of elastin as a main ECM protein in elastic lamina resulted the alteration of vascular structure that affects on vascular function [38, 43].

Regular aerobic exercise can reduce aortic stiffness, regardless of its length or intensity. This complex effect is a result of various physiological responses to exercise, including elevated blood pressure and cardiac output, decreased peripheral resistance and smooth muscle tone with vasodilation, and elevated arterial compliance in the muscular arteries [29]. According to several earlier studies, physical activity under acute settings boosts blood flow and shear stress, which causes an increase in endothelial nitric oxide release [44]. Cross-sectional comparisons show that older people and middle-aged adults who regularly engaged in aerobic exercise exhibit lower aortic stiffness in contrast to their age-matched sedentary colleagues. Higher physical activity is linked to decreased aortic stiffness and pulse pressure in a large population-based sample of community-dwelling older persons. In middle-aged and older persons, aerobic exercise training lowers resting muscle sympathetic nerve activity (MSNA) [30]. In a study with middle-aged and older adults, aortic stiffness was dramatically reduced by systemic adrenergic blocking phentolamine prior to the exercise training intervention, indicating a major chronic regulation of the elastic artery by the sympathetic vascular tone. However, adrenergic blockade had no effect on aortic stiffness following the exercise training. These findings suggest that regular aerobic exercise removes sympathetic adrenergic vasoconstrictor tone that acts on the vasculature. The most potent endothelium-derived vasoconstrictor peptide, endothelin-1, may contribute to the reduction in aortic stiffness brought on by routine aerobic exercise [9, 16, 25].

Our experiment also showed that NO serum is not increase in exercise compared with the control group. Previously, it was found that the change in NO serum was influenced by age with larger effect in older compared with younger subjects [45, 46]. This age-dependent effect was also observed on aortic stiffness when young participants were compared with older participants who conduct aerobic exercise and were measured by PWV [47]. Additionally, the influence of exercise on endothelial function was also affected by the health state of the subject [9]. This means that the more impaired endothelial function, it will be more responsive to exercise training than healthy individuals [9]. This is because it is difficult to improve an already normal vascular function [9]. Our result is also in line with the previous study by Mourot et al. [48] using aerobic exercise performed on a cycle ergometer (30 min, five times a week, with an intensity of 60–70% of the patient heart rate reserve) which did not show any significant increase for nitrate and nitrite concentration after 3 weeks in patients suffering coronary artery disease or heart failure.

We also showed that there is no correlation between NO serum and elastic lamina fragmentation. Although there is no statistical significance, a negative trend showed that the higher the number of elastic lamina fragmentation, there will be a lower NO serum. This is consistent with other findings in our analysis showing that elastic lamina fragmentation is found to be higher in the group with e-cigarette exposure compared with control and without e-cigarette exposure, and additionally, NO serum is lower in the group with e-cigarette exposure. Previous research has found that substrate of NOS such as arginine was significantly correlated with aortic stiffness based on PWV measurement [49]. However, the correlation was weak with *r* = 0.258 [49]. It may be difficult to acquire a significant strong correlation between NO serum and elastic lamina fragmentation because NO mainly influences small arteries compared to larger arteries such as the aorta, and thus, the effect of NO could be small and undetected during the evaluation of the aorta [50].

## 5. Limitation

In this study, we did not examine ROS, which can add value to this research that elastic lamina fragmentation occurs due to oxidative stress processes.

## 6. Conclusion

e-Cigarette exposure has been found to be the cause of elastic lamina fragmentation when compared to the control group and the exercise group. This exposure causes a mark histological structure changes that could indicate a heavier aging process. Aerobic moderate-intensity exercise significantly has less damage than those exposed to e-cigarette but not proven yet to reverse the damage caused by e-cigarette. We also found that e-cigarette decreases NO serum even in the exercise group. Thus, this research shows the histological insight of vascular aging by e-cigarette smoking and how exercise without smoking potentially halt the vascular aging process.

## Figures and Tables

**Table 1:** Differences in the number of layers of elastic fibers in the treatment group.

Group	*N*	Number of layers of elastic fibers			*p* value
		Mean ± SD	Median	Range (min–max)	
K0	7	10.3 ± 1.6	11.0	7.0-12.0	0.144
K1	6	9.5 ± 1.2	9.0	8.0-11.0	
K2	7	9.0 ± 1.0	9.0	8.0-10.0	
K3	6	8.7 ± 0.5	9.0	8.0-9.0	

Description: the Kruskal-Wallis test. K0: control group; K1: moderate-intensity physical exercise group; K2: group exposed to electronic cigarettes; K3: moderate-intensity physical exercise group and exposed to electronic cigarettes.

**Table 2:** Comparison of the number of layers of elastic fibers between the e-cigarette (K2) exposure group and the combination of e-cigarette exposure and moderate-intensity aerobic physical exercise (K3).

Group	*N*	Number of layers of elastic fibers			*p* value
		Mean ± SD	Median	Range (min–max)	
K2	7	9.0 ± 1.0	9.0	8.0-10.0	0.628
K3	6	8.7 ± 0.5	9.0	8.0-9.0	

Description: the Mann–Whitney test. K0: control group; K1: moderate-intensity physical exercise group; K2: group exposed to electronic cigarettes; K3: moderate-intensity physical exercise group and exposed to electronic cigarettes.

**Table 3:** Comparison of tunica intima-media thickness treatment groups.

Group	*N*	The thickness of the tunica intima-media (*μ*m)			*p* value
		Mean ± SD	Median	Range (min–max)	
K0	7	104.8 ± 8.4	92.7	88.0–137.5	0.492
K1	6	95.6 ± 5.1	96.4	80.9–113.7	
K2	7	104.2 ± 4.0	105.6	87.5–121.6	
K3	6	102.2 ± 3.4	101.7	2.0–7.0	

Description: the Kruskal-Wallis test. K0: control group; K1: moderate-intensity physical exercise group; K2: group exposed to electronic cigarettes; K3: moderate-intensity physical exercise group and exposed to electronic cigarettes.

**Table 4:** Comparison of tunica intima-media thickness between groups of e-cigarette exposure (K2) and combination of e-cigarette exposure and moderate-intensity aerobic physical exercise (K3).

Group	Intima-media thickness		*p* value
	*N*	Mean ± SD	
K2	7	104.8 ± 8.4	0.492
K3	6	95.6 ± 5.1	

Note: unpaired *T*-test. K0: control group; K1: moderate-intensity physical exercise group; K2: group exposed to electronic cigarettes; K3: moderate-intensity physical exercise group and exposed to electronic cigarettes.

**Table 5:** Comparison of elastic lamina fragmentation in the treatment groups.

Group	*N*	Elastic lamina fragmentation			*p* value
		Mean ± SD	Median	Range (min–max)	
K0	7	4.6 ± 2.4	4	2-7	0.009
K1	6	2.5 ± 1.9	2	1-6	
K2	7	8.1 ± 2.9	9	4-11	
K3	6	5.8 ± 0.8	6	5-7	

Description: the Kruskal-Wallis test. K0: control group; K1: moderate-intensity physical exercise group; K2: group exposed to electronic cigarettes; K3: moderate-intensity physical exercise group and exposed to electronic cigarettes.

**Table 6:** Comparison of elastic lamina fragmentation between e-cigarette (K2) exposure group and combination of e-cigarette exposure and moderate-intensity aerobic physical exercise (K3).

Group	Elastic lamina fragmentation		*p* value
	*N*	Mean ± SD	
Group			
K0	7	4.6 ± 2.4	0.068^∗^
K1	6	2.5 ± 1.9	
Group			
K0	7	4.6 ± 2.4	0.032^∗^
K2	7	8.1 ± 2.9	
Group			
K0	7	4.6 ± 2.4	0.511^∗^
K3	6	5.8 ± 0.8	
Group			
K1	6	2.5 ± 1.9	0.002^∗∗^
K2	7	8.1 ± 2.9	
Group			
K1	6	2.5 ± 1.9	0.002^∗∗^
K3	6	5.8 ± 0.8	
Group			
K2	7	8.1 ± 2.9	0.082^∗∗^
K3	6	5.8 ± 0.8	

^∗^Mann–Whitney test. ^∗∗^Unpaired *T*-test. K0: control group; K1: moderate-intensity physical exercise group; K2: group exposed to electronic cigarettes; K3: moderate-intensity physical exercise group and exposed to electronic cigarettes.

**Table 7:** Comparison of nitric oxide concentration between groups.

Group	*N*	Nitric oxide concentration (*μ*M)			*p* value
		Mean ± SD	Median	Range (min–max)	
K0	7	44.8 ± 13.2	45.9	23.3-64.9	0.001^∗^
K1	6	55.4 ± 16.2	54.5	30.2-76.4	
K2	7	31.1 ± 8.9	28.6	20.4-46.3	
K3	5	22.6 ± 9.1	21.4	10.5-32.2	

^∗^One-way ANOVA. K0: control group; K1: moderate-intensity physical exercise group; K2: group exposed to electronic cigarettes; K3: moderate-intensity physical exercise group and exposed to electronic cigarettes.

**Table 8:** Comparison between two groups for nitric oxide concentration in K0, K1, K2, and K3.

Group	*N*	Nitric oxide concentration (*μ*M)			*p* value
		Mean ± SD	Median	Range (min–max)	
K0	7	44.8 ± 13.2	45.9	23.3-64.9	0.137
K1	6	55.4 ± 16.2	54.5	30.2-76.4	
K0	7	44.8 ± 13.2	45.9	23.3-64.9	0.049^∗^
K2	7	31.1 ± 8.9	28.6	20.4-46.3	
K0	7	44.8 ± 13.2	45.9	23.3-64.9	0.006^∗^
K3	5	22.6 ± 9.1	21.4	10.5-32.2	
K1	6	55.4 ± 16.2	54.5	30.2-76.4	0.002^∗^
K2	7	31.1 ± 9.0	28.6	20.4-46.3	
K1	6	55.4 ± 16.2	54.5	30.2-76.4	0.0001^∗∗^
K3	5	22.6 ± 9.1	21.4	10.5-32.2	
K2	7	31.1 ± 8.9	28.6	20.4-46.3	0.251
K3	5	22.6 ± 9.1	21.4	10.5-32.2	

^∗^ Unpaired *T*-test with *p* value < 0.05; ^∗∗^ Unpaired *T*-test with *p* value < 0.01. K0: control group; K1: moderate-intensity physical exercise group; K2: group exposed to electronic cigarettes; K3: moderate-intensity physical exercise group and exposed to electronic cigarettes.

## Data Availability

All data are available in the main manuscript.

## Supplementary Materials

Supplementary Materials File 1: author checklist for animal reporting guideline according to ARRIVE guideline 2.0.
